# On Bloodletting

**Published:** 1836-07

**Authors:** 


### PART SECOND.

### Bibliographical Notices.

---

Art. I.—*On Bloodletting.* By James Wardrop, M.D., Surgeon to the late King, &c.—*London*, 1835. Small 8vo. pp. 148.

A treatise on our most important therapeutic agent, by a man of Dr. Wardrop's experience and reputation, is especially entitled to careful critical examination; and our notice of this work will be directed rather to any questionable doctrines which we may deem it to contain, than to those which, by their reasonableness, are calculated to win readily golden opinions from the experienced and intelligent, and certainly in preference to any common-place matters which had become the staple of our craft long prior to their appearance in the present publication.

The work is divided into six *Discourses*, and, from its portions being thus designated, it is probable that they were originally delivered as lectures to pupils, which would account for the elementary nature of a considerable part of their contents. The matter of the first discourse, containing physiological and pathological remarks on the blood, is so strictly of this description, that we shall pass it over without comment; but the more peculiar and practical opinions set forth in the second merit attention.

Local bleeding is the principal subject of this division of the work, and on this point Dr. W. entertains opinions which, if they are not especially his own, he certainly developes more distinctly than any preceding writer. The term "local bleeding" he considers strictly applicable only to blood drawn from the set of vessels which are the seat of the morbid affection: so, at least, we interpret the following passage.

"There are few parts of the body from which blood can be abstracted locally, strictly speaking; for blood taken by leeches or by cupping from the integuments covering a diseased organ, must often come from vessels which are not ramifications of, and have no communication with, the vessels of the diseased organ; the superficial and deep-seated vessels, both arteries and veins, being, in most parts of the body, branches from other trunks. Thus, blood taken from any of the branches of the external carotid, as the temporal arteries, for the treatment of diseases of the head, cannot be considered as a local bleeding, from the distant and circuitous connexion which those vessels have with the internal carotids. Neither, for like reasons, can blood, taken from the parietes of the chest or abdomen, be considered as strictly a local bleeding in affections of the thoracic or abdominal viscera. The opening of a vein in the arm abstracts blood more directly from the jugular veins, as well as from the superior and inferior cava, than leeching or cupping the integuments of the temples, forehead, or those of the thorax or abdomen. What, therefore, has usually been considered as a general bleeding is a more local mode of taking away blood for the treatment of the various affections of the brain, and those of the thoracic and abdominal viscera, than that which is usually resorted to, and commonly considered as a local mode of bloodletting.

"Local or 'topical' bleeding, strictly so called," he continues, "can indeed be only employed in a few organs and in a few parts of the body. In the treatment of diseases within the cranium, topical bleeding can be accomplished by taking blood from the frontal vessels or from the ethmoidal vessels, both of which come from the encephalon, and are branches of the internal carotid artery. It may also be taken from the vessels which pass through the mastoid foramen of the temporal bone, the veins communicating directly with the lateral veins, and the artery being a branch of the occipital which goes to the dura mater." (P. 35.)

That the author regards these distinctions as matters of practical moment we shall assume, in the first place because we hold him in much higher estimation than to suppose he would waste his own and his readers' time in mere verbal quibbles, and, moreover because many passages in his work display the importance he attaches to them. The questions at issue are, first, whether a given quantity of blood drawn by what in ordinary language is termed general bleeding has uniformly more influence over an internal phlogosis than the same quantity taken by local bleeding, employing this phrase to designate the drawing of blood from the integuments corresponding to the organ affected, without reference to the sets of blood-vessels supplying the respective parts; and, in the next place, whether the abstraction of blood from the group of vessels whence the inflamed organ is supplied, possesses the decided advantages which Dr. M. ascribes to it over local bleeding performed in the ordinary way, by cupping or leeching the surface over the part affected.

The author's own remarks on general bloodletting tend considerably to settle the first question.

"It is an excellent rule," he says, "and one which I will venture to say will seldom lead to error, always to employ general in preference to local bloodletting, in such cases of local disease or injury as are accompanied with or have created a disturbance of the general system; whereas, local bleeding ought to be employed in preference to general bleeding, when only local symptoms exist, or when only local symptoms remain after the general symptoms have been subdued by previous general bloodletting." (P. 46.) "The same observations," he adds, "may be made on the treatment of *every disease*, as well as injury, for which bleeding is necessary."

Now it appears to us, that it would have been more consistent with the author's own doctrine to discard local bleeding (general bleeding being the more local thing of the two,) from the treatment of all internal phlogoses, those excepted which are so situated within the cranial cavity as to admit of relief by drawing blood from the Schneiderian membrane, or the integument covering the mastoid process or the frontal bone. In truth, the question is too often treated in the work as if it were one of anatomical research, not of therapeutic observation; and, by floating between the reasoning furnished by the one and the induction from the other, the author is betrayed into inconsistencies. He overlooks what observation has taught us, but what the anatomy of the vascular system does not explain, that in disease there is a connexion—(M. Broussais calls it a sympathy, and we have no objection to this application of the term,)—between the portion of the surface corresponding to certain organs and the organs themselves. This connexion or sympathy is very familiarly exemplified, in cases of internal pain, by the operation of hot flannels, fomentations, rubefacients, epispastics, and (we must add too) of leeches. But, though thus regardless of the principle, derivation, (a principle of acknowledged importance in therapeutics,) that of revulsion finds more favour with Dr. Wardrop. To illustrate its value, he quotes the case of a lady who had suffered during several months from violent cough, accompanied with difficulty of breathing, for which she had applied leeches to the chest, and used a variety of remedies, without effect. Dr. W., being consulted, recommended the application of four leeches to each foot, "and the relief was so speedy and complete that she required very little further treatment." We believe the two principles, derivation and revulsion, which he appreciates so differently, to be in reality very closely related, and that there is no little inconsistency in this respect for the one and contempt of the other. "Derivation," says old Wiseman, "differs from revulsion only in the measure of the distance and the force of the medicines used: if we draw it to some very remote, or it may be contrary, part, we call that revulsion; if only to some neighbouring place and by gentle means, we call it derivation." Notwithstanding our author's general depreciation of ordinary local bleeding, yet we find him, (p. 89,) in place of venesection, applying four leeches to the region of an hypertrophied heart, and thus "subduing both the unnatural vigour of the pulse and the action of the heart."

On the relative value of what Dr. W. calls *strictly local bleeding*, experience must decide: his own estimate of it is very high. Our experience of it, by the application of leeches to the lining of the nostrils in ophthalmia and cerebral affections, has not been inconsiderable; but, although regarding the practice as a very good one, its efficacy has not made us very warm proselytes to the anatomical principle advocated by the author. The surface is a highly vascular one, and, by the application of one or two leeches there, a large quantity of blood is rapidly procured by the suction and the subsequent flow, and unquestionably more relief is afforded than by the same number of leeches to the integuments of the surface; but we think that the rapidity and freedom with which the blood is obtained pretty well explains this superiority, without reference to the distribution of the vessels whence it is drawn.

Our commentary on the third and fourth discourses, in which general bloodletting is discussed, must partake, we regret to say, of the controversial character which a sense of Dr. Wardrop's influence with the public has hitherto compelled us to assume. He selects, in a passage which we have already had occasion to quote, the constitutional disturbance as his guide to the adoption of this measure,—a view in which we cordially concur; but he subsequently qualifies this safe and sound doctrine by annexing the character of the local pain as an additional ground of decision. The indication of that constitutional affection which, he conceives, uniformly justifies and demands general bleeding is incompressibility of the pulse, and he gives the following rule by which it is to be discerned:

> "If, with the point of one finger, the artery be pressed at the wrist, we shall perceive, with another finger applied to the artery beyond and at the distal side of the first finger, that, unless a very considerable degree of pressure be employed, the pulsations of the radial artery will not be entirely destroyed, but the sensation as if of a fine thread or hair will remain." (P. 53.)

All, we believe, who consider this passage, will agree that such an attempt to define the indefinite leaves the question precisely where it was

—in each individual case to be decided by the skill and experience of the practitioner. Were the rule to be acted upon without reference to other circumstances, we apprehend that, in that irritable condition of the system which succeeds hemorrhage, spontaneous or artificial, much mischief might accrue. Laennec's test of the propriety of general bloodletting, though by no means infallible, is infinitely preferable to this.

The expediency of the proceeding being settled, the amount of blood to be drawn is the next subject of consideration. If we are not mistaken in the meaning of our author, his rule on this head is to carry the bleeding *in every case* to syncope; care at the same time being taken, either by the recumbent posture or other means, that the fainting should not occur prematurely; that it should be purely the result of the quantity of blood drawn. We acknowledge that we have experienced considerable difficulty in reaching the author's meaning, and have still perhaps a doubt regarding it. The following passages will probably be thought to furnish some ground of hesitation, and at all events will enable the reader to judge whether we have been successful in interpreting them.

"The state of fainting, which I have already endeavoured to point out as an *unerring criterion* for estimating the extent to which blood should be removed in those cases where general bleeding is most expedient, such as inflammatory diseases attended with febrile disturbance, and in congestions affecting the vital organs, is by most practitioners taken as a guide." (P. 72.)

"The state of fainting *is to be considered as an index* of the quantity of blood which is necessary to be removed for the relief of the disease; and, as I have already said, it will always be found that the quantity is *in the ratio* of the propriety and necessity of abstracting it." (P. 73.)

"But for the abstraction of a certain quantity of the sanguineous fluid in the treatment of diseases requiring depletion, fainting ought *not to be considered* as an *index* of the quantity of blood proper to be withdrawn; but the usual means should be taken to avert its occurrence, except when the patient is in a recumbent posture at the time the venesection is performed. Indeed, when employing bloodletting for the cure of inflammatory diseases, we ought to be particularly cautioned against placing too much confidence in the idea that, if we have bled the patient *ad deliquium*, we have carried the bleeding far enough." (P. 64.)

"It has been laid down as a common rule for the treatment of inflammatory diseases, that blood should be abstracted until syncope is produced, by which we might be led to suppose that the fainting was either a certain token of the quantity of blood that should be taken away, or that it was the act of syncope itself, and not the loss of blood, which was to cure the disease. Hence we often hear of patients being bled until they fainted, as a proof of the vigour of the practice employed, without considering whether the fainting has been produced by excessive depletion or from the particular position of the patient whilst the operation was performed." (P. 63.)

"It is singular how small a quantity of blood some persons can lose: even the very impression on the mind that blood is to be abstracted causes sickness, and fainting comes on after a few ounces of blood have been removed. In such cases, however small the quantity abstracted, there is often a decided relief; the syncope sometimes causing even a permanent subsidence of inflammatory symptoms." (P. 66.)

If our version of these passages is correct, we have no hesitation in saying that, in numerous cases, the adoption of such a rule would produce a waste of blood infinitely worse than useless. We are by no means disposed to advocate a timid practice, always endeavouring to proportion our measures to the exigencies of the case before us, and never stopping the flow of blood till an impression is made on the system; but we should much prefer that this impression were within the limit of syncope than that it attained it. By the adoption of a contrary proceeding, not only in many cases will much more blood be drawn than is requisite, but the necessity for the repetition of venesection will be increased. The degree of reaction which so often occurs after full bleeding has appeared to us to be more general and considerable after complete syncope than where this state has been avoided; whilst, at the same time, the local affection has been aggravated during this secondary excitement: at least, where pain had originally formed a portion of the local symptoms, it has been considerably augmented.

By way of justifying his precepts, Dr. Wardrop gives examples of profuse hemorrhage from various causes, hemorrhoids, rupture of varicose veins, wounds on the field of battle; and he adds,

"Very extraordinary, also, is the quantity of blood that females sometimes lose from the partial separation of the placenta, yet generally without any other bad effect than temporary debility. Women, under such circumstances, have been known to lose several quarts in a few hours. These facts, taken collectively, prove to what an extent blood may be lost without being followed by serious consequences." (P. 79.)

Our author's practice can receive but little justification, we think, from facts of the nature here stated, for, were we to select the circumstance which inflicts most injury on the female constitution, it would be uterine hemorrhage, from whatsoever cause it may arise; and we totally dissent from the opinion expressed of the innocence of profuse sanguineous evacuations in general. Whatever may be thought of certain other points of doctrine which we have taken the liberty to controvert, here at least we look for a decision from the most experienced parts of the profession in our favour.

It is agreeable, after so much controversy, to turn to passages of the work which present no reasonable ground of objection.

In the sixth Discourse will be found some judicious observations on the abuse of bloodletting in apoplexy, and much sound reasoning on the common inflammation which complicates certain specific diseases,—among others, syphilis. The reader will find, too, in the seventh portion of the work, some, though perhaps not very novel, yet certainly judicious, remarks on the use of bloodletting in ulceration. We did not expect that, at this time of day, the author would have found the following piece of information necessary; but as we gather, from the form of expression employed, that it is required in certain quarters, we transfer it to our pages.

"Reflecting on the effect of depletion in such cases, we find incontrovertible proof that sloughing sores are at least not always to be attributed to a diminished vigour or typhoid state of the system, nor to an increased virulence of the specific virus, but to an excess of inflammation arising from the peculiar state of the system or constitution of the patient. The inflammatory state is to be subdued by an antiphlogistic system of treatment, independent of any subsequent treatment which may be necessary for the cure of the specific disease. Hence, as is well known, in cases where the sore shows a disposition to inflame much or slough, the exhibition of mercury is prejudicial. In the cases to which I allude, besides bloodletting, opium ought to be freely given, and one or more grains may be taken every four hours until the symptoms be relieved." (P. 142.)

As a summary of our opinion of Dr. Wardrop's treatise, we are compelled to say that it comprises not a little that is of questionable accuracy, and a good deal of common-place, mixed with much judicious and valuable matter. His talents and experience led us, perhaps, to expect a more philosophic view of the whole question, and that, instead of limiting his researches to the effect of bloodletting on symptoms, he would have endeavoured to ascertain its precise influence over the pathological condition; for in this way only can rules of real value be framed for the application of this, the most heroic of our remedies.

